# Results of WICOVIR Gargle Pool PCR Testing in German Schools Based on the First 100,000 Tests

**DOI:** 10.3389/fped.2021.721518

**Published:** 2021-10-28

**Authors:** Parastoo Kheiroddin, Patricia Schöberl, Michael Althammer, Ezgi Cibali, Thea Würfel, Hannah Wein, Birgit Kulawik, Heike Buntrock-Döpke, Eva Weigl, Silvia Gran, Magdalena Gründl, Jana Langguth, Benedikt Lampl, Guido Judex, Jakob Niggel, Philipp Pagel, Thomas Schratzenstaller, Wulf Schneider-Brachert, Susanne Gastiger, Mona Bodenschatz, Maike Konrad, Artem Levchuk, Cornelius Roth, David Schöner, Florian Schneebauer, René Rohrmanstorfer, Marcus P. Dekens, Susanne Brandstetter, Johannes Zuber, Daniel Wallerstorfer, Andreas Burkovski, Andreas Ambrosch, Thomas Wagner, Michael Kabesch

**Affiliations:** ^1^Department of Pediatric Pneumology and Allergy, University Children's Hospital Regensburg (KUNO) at the Hospital St. Hedwig of the Order of St. John and the University of Regensburg, Regensburg, Germany; ^2^Science and Innovation Campus Regensburg (WECARE) at the Hospital St. Hedwig of the Order of St. John, Regensburg, Germany; ^3^Institute of Laboratory Medicine, Microbiology and Hygiene, Hospital of the Order of St. John, Regensburg, Germany; ^4^Public Health Institute Cham, Cham, Germany; ^5^Public Health Department Regensburg, Regensburg, Germany; ^6^Pediatric Office Judex, Regensburg, Germany; ^7^Maganamed Limited, Regensburg, Germany; ^8^Medical Device Lab, Ostbayerische Technische Hochschule Regensburg, Regensburg, Germany; ^9^Regensburg Center for Biomedical Engineering, University and OTH Regensburg, Regensburg, Germany; ^10^Department of Infection Prevention and Infectious Diseases, University Hospital Regensburg, Regensburg, Germany; ^11^Microbiology Division, Department of Biology, Friedrich-Alexander-University Erlangen-Nuremberg, Erlangen, Germany; ^12^DATEV eG, Nürnberg, Germany; ^13^NOVOGENIA Limited, Eugendorf, Austria; ^14^Research Institute of Molecular Pathology, Vienna BioCenter, Vienna, Austria; ^15^University Children's Hospital Regensburg (KUNO) at the Hospital St. Hedwig of the Order of St. John and the University of Regensburg, Regensburg, Germany; ^16^Medical University of Vienna, Vienna BioCenter, Vienna, Austria; ^17^Intego GmbH, Erlangen, Germany

**Keywords:** children, COVID-19, Germany, PCR, pooling, gargle, schools, pandemic

## Abstract

**Background:** Opening schools and keeping children safe from SARS-CoV-2 infections at the same time is urgently needed to protect children from direct and indirect consequences of the COVID-19 pandemic. To achieve this goal, a safe, efficient, and cost-effective SARS-CoV-2 testing system for schools in addition to standard hygiene measures is necessary.

**Methods:** We implemented the screening WICOVIR concept for schools in the southeast of Germany, which is based on gargling at home, pooling of samples in schools, and assessment of SARS-CoV-2 by pool rRT-PCR, performed decentralized in numerous participating laboratories. Depooling was performed if pools were positive, and results were transmitted with software specifically developed for the project within a day. Here, we report the results after the first 13 weeks in the project.

**Findings:** We developed and implemented the proof-of-concept test system within a pilot phase of 7 weeks based on almost 17,000 participants. After 6 weeks in the main phase of the project, we performed >100,000 tests in total, analyzed in 7,896 pools, identifying 19 cases in >100 participating schools. On average, positive children showed an individual CT value of 31 when identified in the pools. Up to 30 samples were pooled (mean 13) in general, based on school classes and attached school staff. All three participating laboratories detected positive samples reliably with their previously established rRT-PCR standard protocols. When self-administered antigen tests were performed concomitantly in positive cases, only one of these eight tests was positive, and when antigen tests performed after positive pool rRT-PCR results were already known were included, 3 out of 11 truly positive tests were also identified by antigen testing. After 3 weeks of repetitive WICOVIR testing twice weekly, the detection rate of positive children in that cohort decreased significantly from 0.042 to 0.012 (*p* = 0.008).

**Interpretation:** Repeated gargle pool rRT-PCR testing can be implemented quickly in schools. It is an effective, valid, and well-received test system for schools, superior to antigen tests in sensitivity, acceptance, and costs.

## Introduction

Children and youth are still severely affected by the COVID-19 pandemic, even though the acute phase of the disease is mostly mild in the young (1). They are over-proportionally affected by secondary consequences of the pandemic such as social deprivation, lack of physical activity, decrease in economic status, and dysconnectivity, especially in rural communities (2, 3), and in countries like Germany, where closing of schools was not perceived as the last option in fighting the pandemic but as the first (4, 5).

Consequently, severe psychological and developmental impairments have now become obvious (6). On the other hand, SARS-CoV-2 infections may also lead to major health problems in children in the long run (7, 8): Pediatric Inflammatory Multiorgan Syndrome (PIMS) is a severe, potentially deadly consequence of COVID-19, affecting only the young (9). Children are also affected by post-COVID syndrome (PCS). Therefore, it is of the utmost importance to balance the needs of children to attend school and have a chance for social development despite the pandemic, with the proper protection to minimize the risk of SARS-CoV-2 infection in the school environment (10). In the current state of the pandemic, such concepts cannot wait but need to be implemented now (11).

We gained experience in a proof-of-concept study, which started in the summer of 2020, on how testing of school children can be achieved and contribute to safety in schools (12) in addition to already existing non-pharmaceutical interventions such as wearing face-masks, maintaining social distance, disinfecting hands, and increasing ventilation in rooms, all of which were implemented in German schools in the autumn of 2020. Based on this experience, we developed a safe, efficient, and cost-effective SARS-CoV-2 testing system for schools: WICOVIR (Where Is the COrona VIRus?). Here, we present the concept and provide the first data based on >100,000 tests. Due to the introduction of compulsory antigen testing in schools in Bavaria starting on April 12 (week 15), 2021, we had the opportunity to compare self-administered point of care (PoC) antigen tests to gargle pool rRT-PCR tests for 6 weeks.

## Materials and Methods

### Study Design and Population

**Figure 1:**
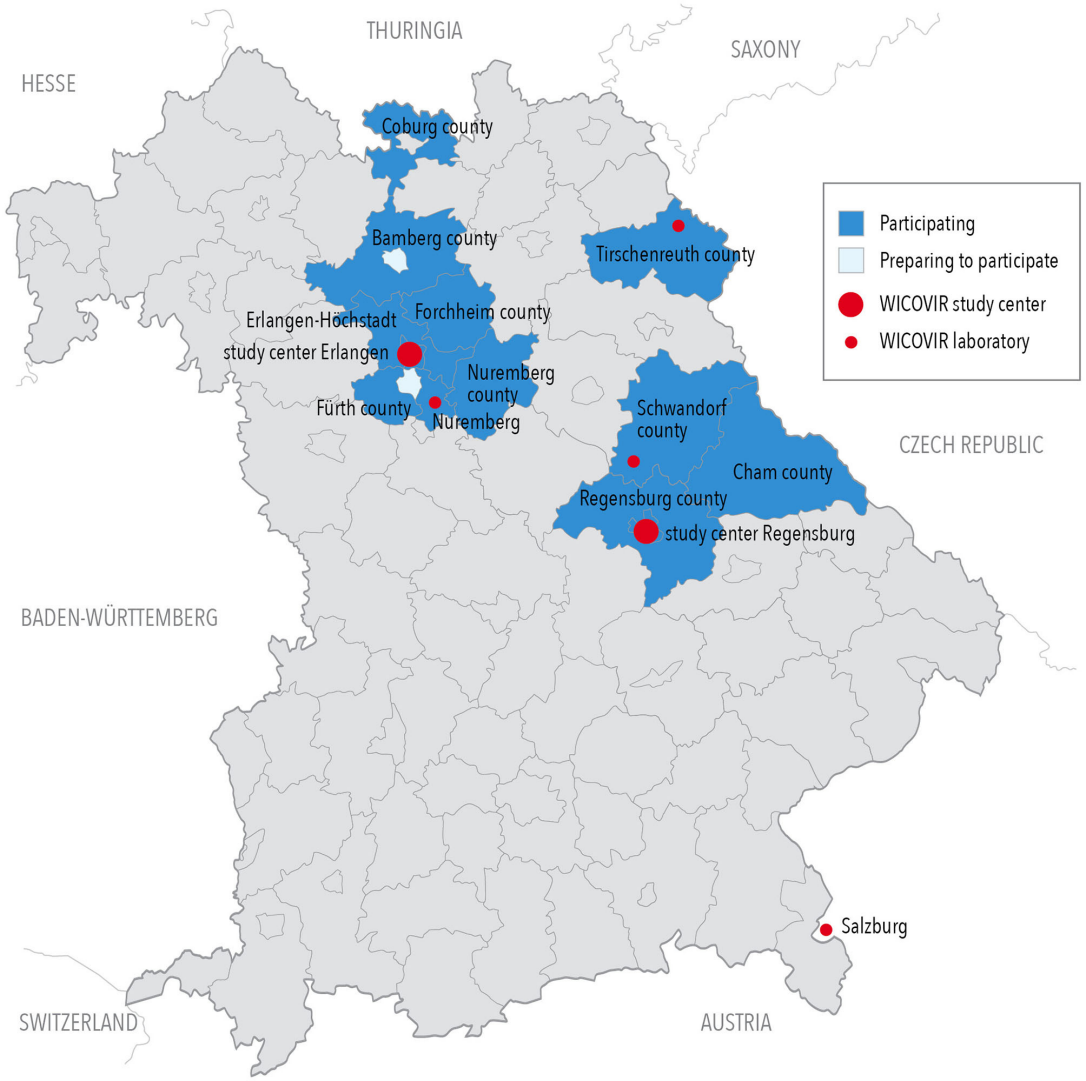
Map of Bavarian counties participating in WICOVIR as of May 15, 2021.

**Figure 2:**
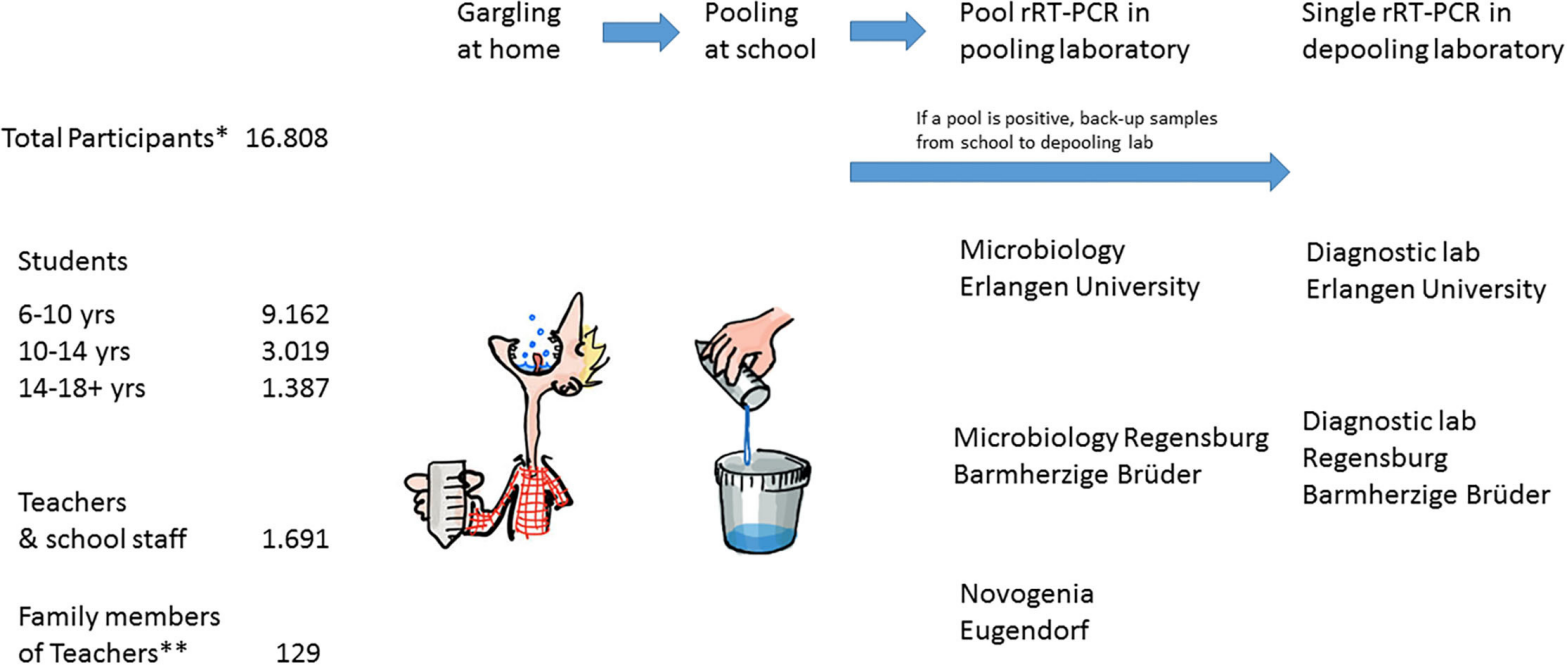
Study design and numbers of the WICOVIR study as of May 12, 2021. *Participant numbers change over time, as new schools were included and some schools/individuals may have dropped out while others joined. **In pilot the phase, we explored teacher pools (as no clearance for student tests was yet available) and allowed family members of teachers to participate in these teacher pools.

The objective of this proof-of-concept study was to show that regular gargle pool rRT-PCR testing is safe, efficient, and cost-effective in all school environments, including students from first grade (~6 years of age) to grade 12 (~17 years of age) of all German school forms. Here, we report on our experience after 11 full school weeks (and 2 weeks of vacation) of testing. After achieving approval from the Bavarian State-Ministry for Education and Cultural Affairs (February 26, 2021), and funding from the Bavarian State-Ministry for Health and Medical Care (March 26, 2021), we started the pilot phase, which lasted for 5 full school weeks and 2 vacation weeks to build up the test system and which was followed by 6 weeks of the main study phase of regular testing after Easter vacation. We invited all schools in counties close to the two original study centers in Erlangen and Regensburg to participate in the study through internet platforms, print media, and personal information (Figure 1). Interested schools were asked to participate in two introductory webinars taking place twice weekly, where the study design was explained (Figure 2). Detailed information material was developed for the study, specifically addressing the information needs of children, youth, parents, and school staff. These were made publicly available through the study website (www.we-care.de/WICOVIR).

Schools that participated had to agree to study terms, e.g., to comply with hygiene standards and study protocols and a data protection contract had to be signed. Through participating schools, informed consent was obtained from parents, school children, and staff who volunteered to participate in the project. The prerequisite for participation was informed consent and school attendance; the exclusion criteria included a positive SARS-CoV-2 test result within 2 months prior to participation (to avoid positive results in rRT-PCR testing due to prolonged viral RNA shedding not indicating infectivity).

Due to the specific conditions during the third wave of the pandemic, we distributed study information by digital channels/website, FAQs, emails, and phone calls to address all questions of participants. The participation in the study was voluntary. For reasons of anonymization, communication with study participants in the course of the study was through the schools only. We trained teachers and school staff in study procedures through on-site initiation visits. Transport of samples was organized through schools and voluntary helpers, or, if that was not possible, through a courier service or study personal. A drive-through to make sample delivery easy for volunteers was established outside the laboratory. We also established a network of primary care pediatricians who volunteered to support schools in all questions concerning the study and infection protection in case of positive results. The study was approved by the Ethics Committee of the University of Regensburg (file-number: 21-2240-101).

### Data Collection and Management

**Figure 3:**
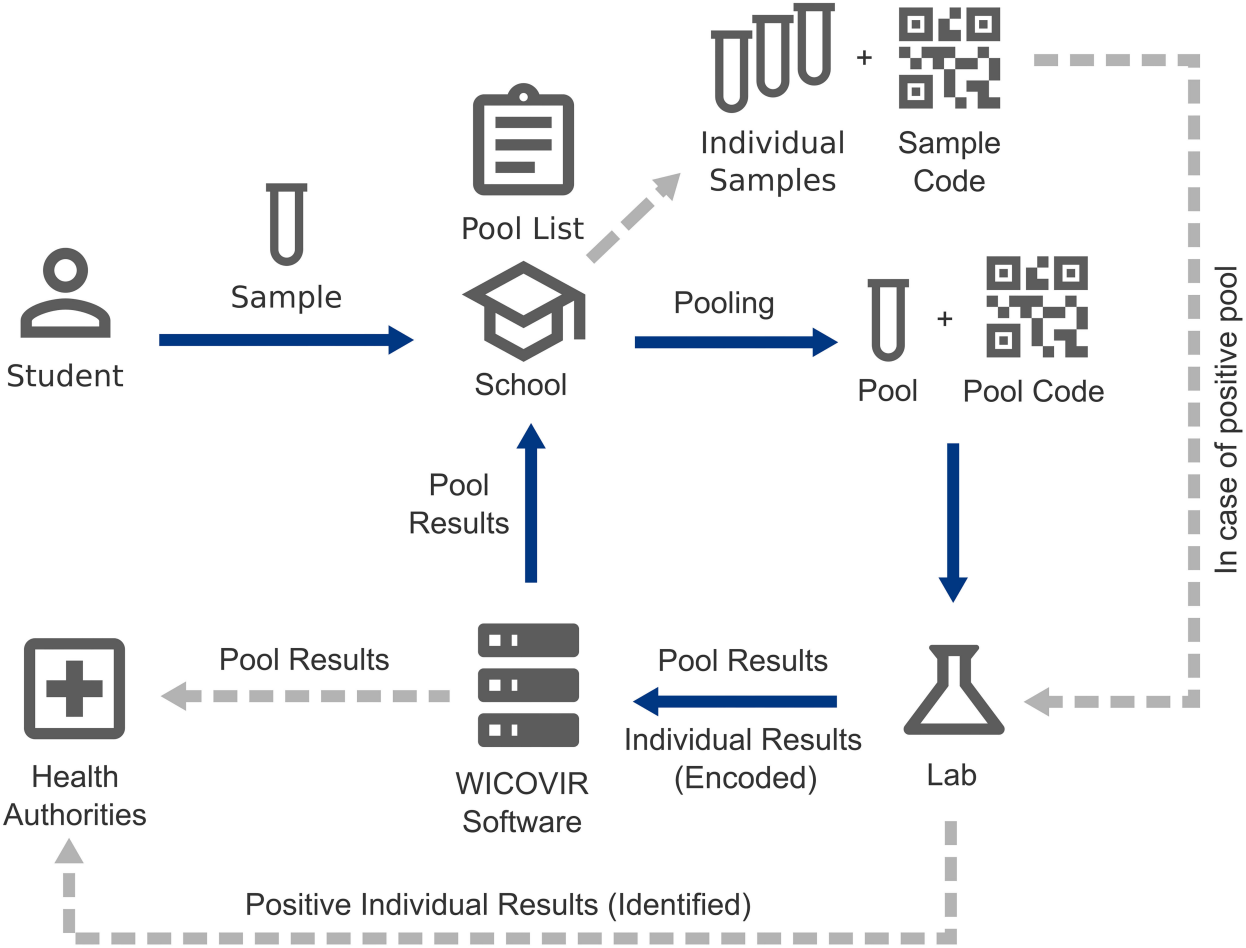
Sample/data flow and data protection. Student samples were pooled in the participating schools, who kept analog records of pool participants and registered pools in the software. Pools were barcoded and sent to the lab. Pool test results were documented in the software by the lab and schools received results through the software. In the case of a positive pool, individual samples pertaining to a positive pool were sent to the lab and individual results were entered into the software in a pseudonymized fashion. Health authorities had access to the pseudonymized data in the software and received personalized results directly from the labs through official reporting software.

The data protection principle of the study was to collect as little data from participants as possible. No personal or medical data of participants were collected in the pooling study. Only the schools kept track on-site of who participated in a specific pool. Those records were deleted within 24 h and were only needed to resolve positive pools. A browser-based software tool was developed for the study by MaganaMed GmbH to keep track of barcoded pools, pool results, pool dissolving, and to allow for automated correspondence of test results and summary statistics of test results, irrespective of the laboratory software in the participating test centers. The software only handled pool IDs and alphanumeric sample IDs (unique, pseudonymized), but no personal information on participants. All identifying information was exclusively handled by participants, schools, diagnostic labs, and health authorities, respectively (Figure 3). Additional information on the software is available upon request from the authors or from the company (https://maganamed.com).

### Gargle Procedures

The feasibility of gargling (throat washings) for SARS-CoV-2 detection has been shown previously (13). Even though the diagnostic sensitivity is slightly lower when compared to nasopharyngeal swabs, the absence of invasiveness of gargling is a decisive advantage, especially in our setup of repetitive testing in children. In this study, all participants gargled with ~6 ml of tap water at home twice or three times per week, first thing in the morning (before brushing teeth and breakfast) for ~30–60 s to achieve maximal recovery of virus from throat rinsing. Feasibility of the gargling procedure in the school setting was tested previously in the STACADO study and reported elsewhere (12). A video providing exact guidance and documentation of the gargling procedure is available online at www.we-care.de/WICOVIR. Gargle recovery fluid was collected by the participant in a screw-cap tube and divided into a second screw cap tube in approximately equal amounts (2–3 ml each). Both tubes were brought into school in a zip-lock bag. One was for pooling and the other one (back-up) was retrieved from schools and tested only in the case of a positive pool result.

### Pooling Procedures

In the schools, one tube was emptied by the participant into a pooling container that was positioned in a pooling station. Pool participants were defined by the schools and usually contained the pupils of one class and the school staff (teachers) attached to that class. The maximum number of participants accepted for one pool was 30. In the Erlangen study site, we explored testing in pools of teachers with their attached families in a small set-up including 129 family members in teacher-centered pools. The pooling station was specifically designed by the Medical Device Lab of OTH Regensburg (Ostbayerische Technische Hochschule Regensburg) for the purpose of this study according to exact hygiene specifications developed to avoid splash contamination. Prototypes were provided by the technical workshop of the University of Regensburg. Pooling stations were manufactured according to our specifications and donated to the study unconditionally by local industry (Krones AG, Regensburg, Germany). A video documenting the pooling procedure is also available at the study website (www.we-care.de/WICOVIR). In brief, pooling took place under the supervision of a teacher in classes, and schools defined and documented participants of their pools in-house. Only the number of participants in a pool was transmitted for data protection reasons. Every sample contributing to a pool was defined as a test sample. After pooling, the pooling containers were sealed and transported to the laboratory within 1 h.

### Depooling Procedure

The second tube (back-up tube) with gargle fluid was kept with the students/at school and was only retrieved in the rare event of a positive pool. In that case, back-up tubes of all participants in a positive pool (according to the documentation of the school) were barcoded with a unique identifier at the school so that only pseudonymized samples were transported to the medical laboratory which provided the individual medical testing of samples by PCR procedures certified for medical testing. In cases defined as urgent by the public health authorities, schools were requested to provide clear names to the laboratory immediately in accordance with the infection protection act. For all samples in the Regensburg region, depooling was achieved within 12 h after pool samples entered the laboratory; for all other cases, this was achieved by at least the next day.

### SARS-CoV-2 Pool rRT-PCR Testing

To test gargle pools, we applied previously described (14, 15) as well as recently optimized methods. As WICOVIR is a proof-of-concept study for the rollout of a pool test system in the state of Bavaria, we allowed for different, site-specific rRT-PCR methods, to test if already existing laboratories could be integrated in a large-scale rollout. Individual gargle samples of known virus content were used to determine detection limits in different pool sizes with the different methods. All test methods were able to detect a positive sample with a set cycle threshold (CT value of 32 in a pool of 30 samples). We performed conformation tests between sites and laboratories. Specifically, we continuously tested positive pools in different labs in ring experiments (data available on request). Analytical methods for pool rRT-PCR of the different laboratories are shown in Table 1 and given in detail in the Supplementary Material section.

**Table 1:** Comparison of PCR test methods in WICOVIR laboratories in this study phase.

**Test steps**	**Regensburg**	**Eugendorf/Salzburg**	**Erlangen**
Sputolysis	**–**	Ascorbic acid	Ascorbic acid
RNA isolation	RNA extraction:	RNA extraction:	Lysis:
	MagNA Pure DNA/RNA kits (Roche)	MagnifiQ RNA buffer kit (A&A Biotechnology)	Tris(2-carboxyethyl)phosphine hydrochloride (TCEP HCl) (Sigma-Aldrich)
	BEXS Ready Viral DNA/RNA kits (Inno-train)		
	MagnifiQ RNA buffer kit (A&A Biotechnology)		
qPCR master mix	LightCycler® Multiplex RNA Virus Master (Roche)	FTD™ SARS-CoV-2 (Siemens Healthineers)	2 × Luna Probe One-Step Reaction Mix (NEB)
Targeted genes	E gene of SARS-CoV-2	N gene and ORF1ab region of SARS-CoV-2	N1 region of the N-gene of SARS-CoV-2
Extraction control	Equine arteritis virus (EAV)	Equine arteritis virus (EAV)	RNAse P
PCR cycler	Light Cycler 480 II (Roche)	Quantstudio 5 (Thermo Fischer Scientific)	qTOWER^3^ G (AnalytikJena)
Confirmation method	Xpert Xpress™ SARS-CoV-2 assay targeting E and N2	Initial assay already targets 2 genes	N1 and N2 regions of the N-gene of SARS-CoV-2

### Online Survey on Acceptance of Test Regimes in Schools

To assess the acceptance of the WICOVIR gargle pool rRT-PCR and self-administered antigen tests, we designed an anonymous online survey applying our previously reported qnome database and questionnaire system (www.qnome.eu). The questionnaire consisted of 15 questions, could be used freely, and is available upon request. All school heads of participating schools (*n* = 96) at the time point of the survey (week 3 of the main phase) were invited to fill out the questionnaire, as both the WICOVIR testing and the antigen PoC tests were performed concomitantly in these schools, allowing for direct comparisons of the procedures.

### Statistical Analyses

Data from the gargle pool tests are presented using descriptive statistics. For analyzing the difference between proportions of positive tests between different phases of the study, statistical tests that considered dependent groups could not be performed (as individuals with repeated measurements were included but anonymized); thus, we performed the non-parametric Mann–Whitney *U*-test. Differences in the data from the online survey assessing indicators of acceptance were analyzed using a *t*-test for dependent groups for metric indicators and McNemar tests for dichotomous and dichotomized indicators. All analyses were performed using SPSS.23.

## Results

**Figure 4:**
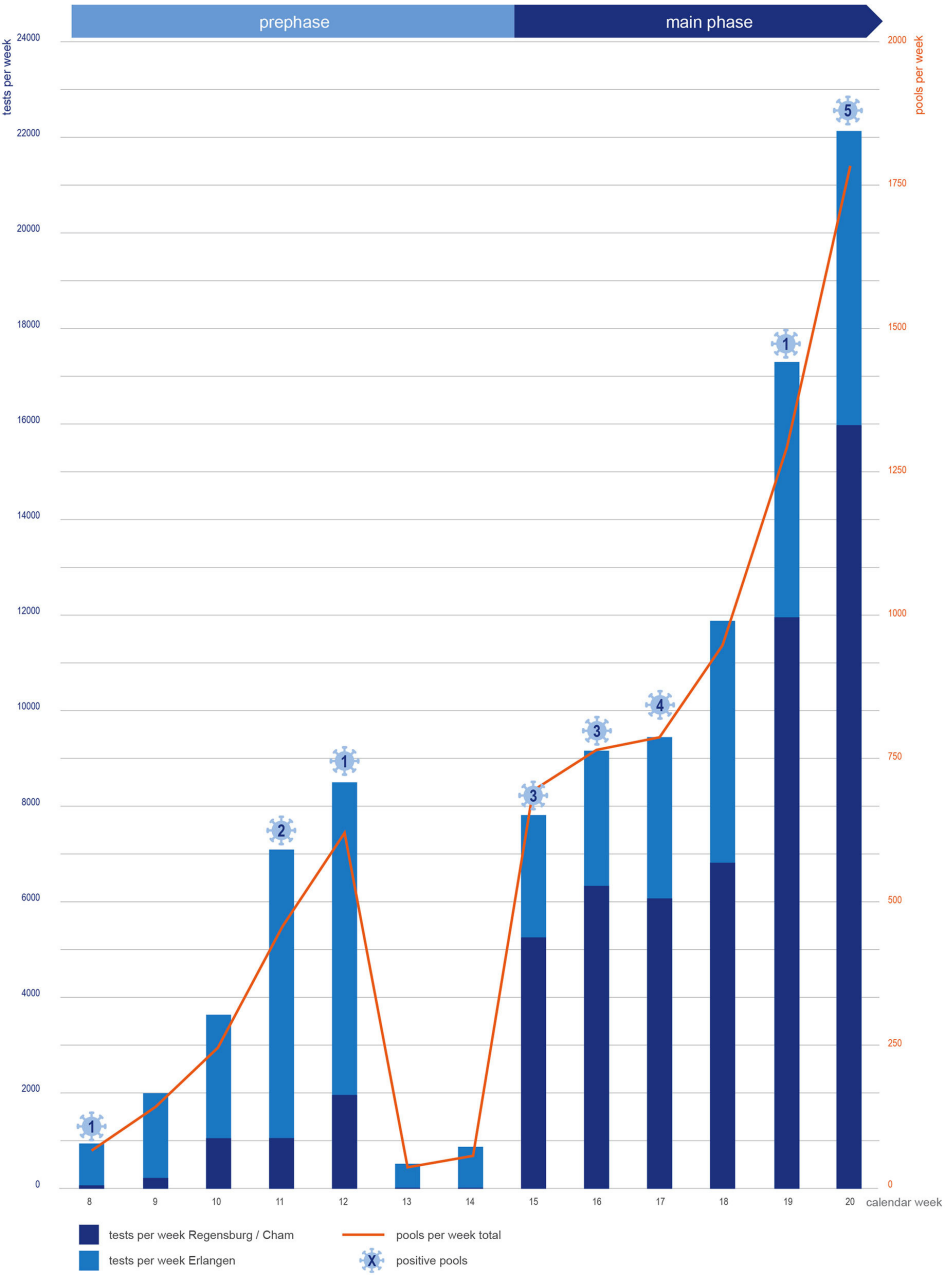
Development of test and pool numbers in the pilot and main phase of the WICOVIR project and positive results of testings per week.

We performed 23,582 tests pertaining to 1,621 pools in the school setting in the pilot phase of the study and the adjacent vacation (Figure 4 and Supplementary Table 1, upper panel) to establish all study procedures, test feasibility, and acceptance of methods. The pilot phase lasted until students returned to schools after Easter vacation and the main study phase started on April 12. In the main phase, 114 schools participated. In total, 16,808 individuals participated, and of these, 14,988 were students of different age groups (Figure 2) The main study phase, which has lasted 6 weeks so far, started with three laboratories that provided regular pool testing (Erlangen, Regensburg, and a diagnostic laboratory in Eugendorf, as capacities in Regensburg could not be ramped up fast enough to cover the demand in the initial phase). Depooling using the back-up samples was performed in Erlangen and Regensburg (for pools tested in Regensburg and Eugendorf). In the main phase, we performed 77,763 tests in 6,274 pools, with an average of 12,800 tests per week and an average pool size of 13, respectively (Supplementary Table 1).

Within the pilot phase, we identified four positive pools, and 16 positive pools were found in the main study phase (Table 2). The average CT value of a positive pool was 34 (range 26–39), and it contained a mean of 14 tested individuals (range 4–26), which corresponded to an average CT value of 31 (range 24–37) in the back-up sample of individual positive pool participants. In these 20 positive pools, we detected a total of 19 novel infections. In the Regensburg study center, three already known cases of previous SARS-CoV-2 infections in children who still underwent testing as requested by the study protocol were identified. In the Erlangen study center, where also relatives living in the same household were invited to take part in the testing, two positive pools showed two positive individuals each. Also in Erlangen, one pool could not be resolved successfully as not all back-up samples could be retrieved reliably in the pilot phase of the study. Of those that were found to be positive, all but two were students. Overall, we found a positive rate of 1:400 in pools, respectively, translating to one newly identified positive individual every 5,600 tests.

**Table 2:** Characteristics of positive pools and positive individuals in the WICOVIR project.

**ID**	**Status**	**Pool size**	**CT Pool**	**CT single**	**Antigen test**	**Comment**
1	Student	15	33	32	Not available	COVID-19 residue
2	Student^*^	26	32	37	Not available	
3	Teacher's husband^*^	26	32	30	Same day, positive (after pool result)	
4	Student	8	34	30	Not available	
5	?	7	34	?	Unknown	Not all single samples retrieved
6	Student	14	36	31	Same day, negative	
7	Student	20	36	36	Next day, negative	
8	Student	9	39	36	Same day, negative	
9	Student	8	34	36	Same day, negative	
10	Student	14	32	27	Same day, negative	
11	Student	6	35	28	Not available	
12	School staff	12	33	32	Same day, positive (after pool result)	
13	Student	15	35	34	Not available	COVID-19 residue
14	Student	13	35	33	Not available	
15	Student+	20	34	27	Same day, negative	
16	Student+	20	34	32	Same day, negative	
17	Student	8	34	27	Same day, negative	
18	Student	9	34	26	Same day, positive	
19	Student	4	26	24	Not available	
20	Student	13	36	29	Same day, negative	
21	Student	10	39	36	Not available	COVID-19 residue
22	Student	20	33	31	Not available	

* *and ^+^ mark positive individuals from the same positive pool*.

In schools that participated in the pilot phase, voluntary participation rates of students were between 95 and 98%. Compulsory antigen testing was introduced in Bavarian Schools on April 12, 2021; however, children participating in the WICOVIR project were allowed to continue the WICOVIR test regime by law under the condition that they perform one antigen test per week (usually at the first day of the week present at school) to assure that the WICOVIR procedure was safe. That gave us the unique and unexpected opportunity to compare sensitivity of compulsory self-antigen tests to WICOVIR gargle pool rRT-PCR testing at a large scale: On every Monday morning from calendar weeks 15–20 (main phase), all children participating in WICOVIR testing that day also had to perform antigen tests concomitantly (leading to a total of ~25,000 concomitant tests). Out of eight antigen tests that were done in schools on the same morning that gargling was also performed, all but one showed negative results as did one antigen test performed the day after the positive PCR result (Table 2). Twice, antigen tests showed a positive result when applied for confirmation after pooling and depooling had already identified a positive individual. Based on these data, we calculated sensitivity for the early stage of the infection in the school setting of self-administered antigen tests to be 12.5% (1/8) to 27.3% (3/11) compared to the truly positive results by pool rRT-PCR tests. We cannot calculate the sensitivity and specificity of gargle pool rRT-PCR in this setting, as no more comparable sensitive testing was performed to define sensitivity and no positive cases outside the WICOVIR testing were reported to be found.

Three weeks into the main phase of the project, we noticed a decrease of positive results (weeks 15–17: 0.042% vs. weeks 18–20: 0.012%; *p* = 0.008). Interestingly, positive cases also in the last 3 weeks were restricted to children who joined the testing system for the first time within the 2 weeks before.

**Figure 5:**
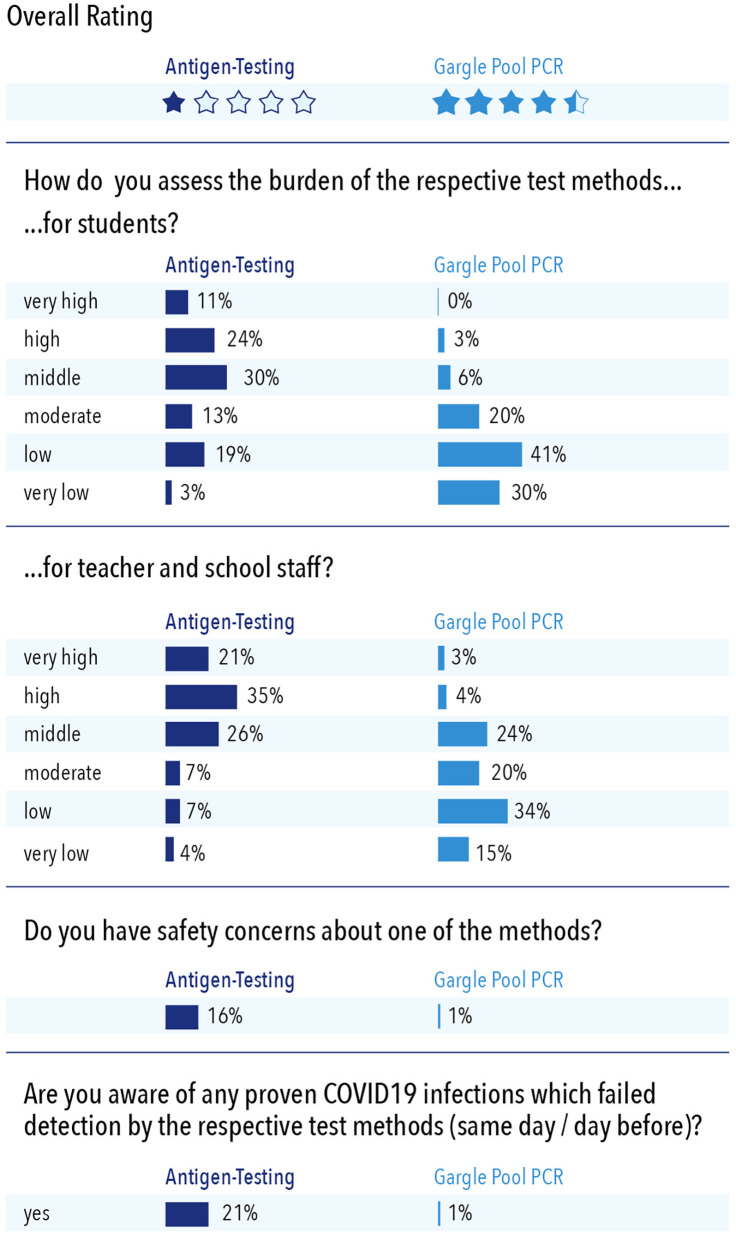
Indicators of acceptance of antigen tests and gargle pool PCR tests in schools based on 71 responses from school heads (74% participation rate).

Three weeks after the WICOVIR main phase and the compulsory self-administered antigen testing had started, we invited all schools that performed both concomitantly to give anonymous feedback in an online questionnaire on their experience (*n* = 71 of 96 invited school heads responded). Significant differences in acceptance, handling, and overall ratings were observed for both procedures (Figure 5). Overall, gargling was received significantly better than antigen testing, resulting in an overall “school grade” of 1.5 for gargle pool rRT-PCR tests compared to 4.1 for antigen tests (grades 1–6, where 1 is best).

## Discussion

Repeated gargle pool rRT-PCR testing can be implemented quickly in schools as shown in our WICOVIR project. It is an effective, valid, and well-received test system for schools to detect SARS-CoV-2 infections in a rather early phase with high CT values. According to our data, it is superior to antigen tests in sensitivity and acceptance.

Repeated testing of large parts of a population is thought to be a major public health tool against the COVID-19 pandemic (16). Testing becomes especially important, when other measures of protection (such as vaccination) are not available or not feasible (such as complete social isolation) for a population, as is the case (and will be for quite some time) for children. Models estimate that, in theory, testing 75% of a population twice a week with a fast turnaround of reliable test results and immediate protection measures will break infection chains and contribute, together with other measures, to a “no-COVID situation” within 4–6 weeks (16). To achieve high testing frequency, testing needs to be extremely cost-efficient and easily scalable. Gargle pools do not require additional staff for swabbing. Furthermore, pooling in schools helps to drastically reduce the number of samples to be handled in the laboratory (by a factor of >10).

While being the most accurate method, individual rRT-PCR testing is still the most expensive diagnostic procedure. PoC antigen tests are cheaper, are much less sensitive, and require professional swabbing. No costs for swabbing occur in the case of self-applied antigen tests, but since those tests are restricted to sample collection from the front part of the nose by the children themselves, sensitivity of those tests may be diminished in comparison to professional swabbing. Gargle pool testing considerably reduces costs for an individual test, mainly for three reasons: (1) Depending on the pool size, pooling itself reduces costs by a factor of 10–30. At no point in our study did we find any indication that, in a realistic set-up, pool sizes of up to 30 participants would limit detection sensitivity. (2) Gargling does not require any staff for swabbing, which drastically reduces costs for sample collection (for German PoC tests, two-thirds of the PoC costs come from sample collection). (3) From a formal point of view, gargle pools are considered to be a preemptive public health test, but have no individual medical diagnostics. As a consequence, they may be performed outside of medical labs. Thus, we have found that gargle pool testing can be provided at an overall cost (including transport, personal, equipment, and consumables) of <1 EUR per person tested.

To offer a widespread testing of school children, testing needs to be simple but sensitive, acceptable for the tested child and their parents, readily available, and easily accessible. All current standard test systems are lacking one or another quality needed for such a broad test regimen. As we aimed to establish such a system in schools, we first addressed the questions of test acceptability. We had already gained experience with gargle rRT-PCR tests, which were introduced as the testing standard in our university children's hospital for clinical practice and study purposes in March 2020. When compared to nasopharyngeal swabs, only a slight decrease in sensitivity was observed for gargle samples (13). We found a high acceptance rate of these tests in our STACADO and STACAMA studies in children (12). The youngest children that can perform gargle maneuvers in our clinical setting were 3 years old, and as a general rule, children who can brush their teeth themselves can also gargle. In those studies, children gave very clear feedback, that they (and in some cases even the parents) rejected gargling with physiological NaCl (0.9%). Therefore, we introduced gargling with distilled water and later with tap water (or still mineral water), neither of which interfered with SARS-CoV-2 PCR testing. In the STACADO setting, we had started with gargling at school but quickly it became obvious that the procedure was so simple that it could be performed at home without losing quality with the advantage that the yield of potential virus material was expected to be higher when the specimen was sampled first thing in the morning due to reduced airway clearance during the night (17). Aspiration risk with such low quantities as 6 ml of water is neglectable. Thus, gargling is a safe, painless, easy-to-perform, and robust method to collect repeated samples in children.

However, gargling at home has the disadvantage that samples are not collected under supervision and study procedures may not have been performed perfectly in the home setting. Thus, this is a limitation of the procedure. When the samples are pooled in the school, it can usually be determined easily if gargle fluid is in the tube (in comparison to clear water) and if the amount of the gargle sample is as expected. When we performed quality control in random pools, all tested single samples contained human RNA as an indicator that gargle fluid had been collected. However, it is expected in this test system, like in all others except a professional swab taken by trained medical personal, that a perfect probe cannot be guaranteed. Furthermore, the acceptance of the tests according to the results from our online survey with school heads and by the families according to voluntary participation rates of 95–98%, was surprisingly good, suggesting that gargling is a feasible procedure in children.

rRT-PCR pool tests were established for SARS-CoV-2 testing early on in the pandemic (18) and further developed by members of our consortium (14) as well as compared systematically to other techniques (15). In our study, the average CT value for a positive pool was 34 and that of the individual positive sample in that pool was 31. Thus, in most cases the positive individual was detected so early that passing on the infection in the school environment with hygiene concepts in place was rather unlikely based on what we know currently and what we observed in WICOVIR.

The challenge in a school setting is the timely performance of the pooling and the subsequent testing that provides a great challenge to routine laboratories together with the organization of depooling in the case of a positive pool and the communication of results when pools are used. We have solved all these issues in WICOVIR. Pooling is performed in the schools using pooling stations to speed up the process (and to reuse the gargle tubes, overcoming the issue of limited supply of plastic ware, and reducing the plastic waste in the pandemic). We keep personal data of participants only in schools and no personal data go to the lab with the pooling container. Pool testing is thus anonymous but can direct true individual testing to where the virus is to be found, saving resources as recently published (19). The drawback of this anonymous testing in WICOVIR is that we cannot evaluate population characteristics of the total test population, except for those few that tested positive.

Our results show how superior in sensitivity gargle pool PCR testing is compared to antigen tests. Only gargle pool rRT-PCR detected nine true positive cases in ~25,000 tests when both gargle pool rRT-PCR testing and self-administered antigen tests were applied the same morning compared to one positive antigen test. At this stage, gargle pool rRT-PCR testing as applied here did not show false-positive or false-negative test results to the best of our knowledge. However, with increasing number of tests, we expect to also find rare cases of false results with this system as with any other testing.

Furthermore, two antigen tests, performed after the positive PCR result was already available, were positive. It has to be noted that antigen tests are specifically not designed to detect early SARS-CoV-2 infection (20, 21). This difference becomes especially obvious if testing is performed repetitively, when in most cases gargle pool testing can prevent infection cascades in schools while antigen tests cannot. According to the health authorities in the County of Cham, where all primary school children (~*n* = 4,200) in 38 schools participated regularly in WICOVIR by default, no SARS-CoV-2 infection was detected in study participants outside the WICOVIR testing, suggesting a very high sensitivity of the pool rRT-PCR performed in the study.

Overall, we detected 19 novel infections by our school test system. In the fourth and fifth week of the main phase, only 1 of the more than 27,000 tests within those 2 weeks was positive, suggesting that repeated tests make the group more safe, especially as the one positive individual during that period had just joined the test system with a first (positive) test. However, also the general incidence of SARS-CoV-2 infections in the participating counties dropped at the same time. When compared to the incidence of the counties participating in the tests (incidence of 100–250 per week), we found that children in schools were positive less often than expected (1 out of every 5,600 tests) while at the same time, children and youth seem to contribute to the disproportionally strong overall incidence according to RKI data^1^. This leads to the conclusion that they get infected anywhere but in the schools, e.g., in close contact with positive family members, relatives, and friends outside the schools. Accordingly, no indication for a large number in school children was found.

Prior to our studies, we were unsure whether a high SARS-CoV-2 infection rate in the general population would limit pool sizes and increase costs. From a practical point of view, this has never been problematic in any of our regions under observation, even with an incidence of up to 250 new infections per 100 k people in 1 week. One reason for this may be that individuals tested in our settings have typically been non-symptomatic, which is different from other testing set-ups such as emergency sites at hospitals or local testing centers. Specifically, school children and students with symptoms were requested by a directive of the ministry, implemented in February 2021, to present to the local pediatrician, stay at home, and not attend school before tested negatively.

We conclude after >100,000 tests that gargle pool rRT-PCR testing is an easy, sensitive, and robust test system for schools. Especially as children in primary school will not be vaccinated any time soon, such a smart and suitable test system for children that can be implemented easily is urgently needed and shall be rolled out immediately. Our data show that with a proper testing concept in place, schools are a safe place for children in times of the pandemic.

## Data Availability Statement

The raw data supporting the conclusions of this article will be made available by the authors, without undue reservation.

## Ethics Statement

The studies involving human participants were reviewed and approved by Ethical Committee of the University of Regensburg. Written informed consent to participate in this study was provided by the participants' legal guardian/next of kin.

## Author Contributions

MKa, AA, AB, DS, and TWa contributed to conception and design of the study. PS established and supervised the field work, which was performed by PS, BK, HB-D, EW, and SGr. AB, AA, and DW organized and supervised the laboratory work. PP, AB, MD, and JZ developed novel methods. PP, MA, EC, TWü, HW, SGa, MB, MKo, FS, AL, CR, and RR performed laboratory work. MG, JL, and BL performed contact tracing and public health measurements. JN and PP provided IT support and developed the software for the project. GJ organized and supervised support of local pediatricians. WS-B supplied the hygiene concept and performed sequencing. TS developed the pool testing devices. SB performed the statistical analysis. PK wrote the first draft of the manuscript. AA, AB, DW, and TWa wrote sections of the manuscript. MKa wrote the final version of the manuscript. All authors contributed to manuscript revision, read, and approved the submitted version.

## Funding

This work was supported by the Bavarian State Ministry of Science and Arts (Grant STACADO) and the Bavarian State Ministry of Health (Grant WICOVIR) and funds from the charity of Blaue Schwestern.

## Conflict of Interest

TWa has invested in a company that performs PCR pool tests for companies. RR, FS, and DW are employed by NOVOGENIA, a commercial PCR test laboratory. JN and PP are employed by Maganamed GmbH, a commercial software company. DATEV eG is a software company which provides a non-profit test center for the project. The remaining authors declare that the research was conducted in the absence of any commercial or financial relationships that could be construed as a potential conflict of interest.

## Publisher's Note

All claims expressed in this article are solely those of the authors and do not necessarily represent those of their affiliated organizations, or those of the publisher, the editors and the reviewers. Any product that may be evaluated in this article, or claim that may be made by its manufacturer, is not guaranteed or endorsed by the publisher.

## Footnotes

^1^ https://experience.arcgis.com/experience/478220a4c454480e823b17327b2bf1d4
