# Supplementary material for: Results of WICOVIR Gargle Pool PCR Testing in German Schools Based on the First 100,000 Tests
Source: Front Pediatr. 2021 Oct 28;9:721518. doi: 10.3389/fped.2021.721518 (PMC8581236; doi:10.3389/fped.2021.721518)
Supplement: Supplementary file 1 [file Table_1.DOCX]

**Supplemental Table 1**: Pool and test numbers as well as positive tests per center per calendar week

| **Regensburg Center** | | |  |  | **Erlangen Center** | |  |  |
| --- | --- | --- | --- | --- | --- | --- | --- | --- |
| **week** | **tests** | **pools** | | **positive pools** | **tests** | **pools** | **positive**  **individuals** | **comments** |
| W 8 | 69 | 5 | | 1 | 876 | 62 | 0 |  |
| W 9 | 225 | 20 | | 0 | 1772 | 122 | 0 |  |
| W 10 | 1056 | 66 | | 0 | 2585 | 178 | 0 |  |
| W 11 | 1059 | 90 | | 0 | 6039 | 358 | 2* | *2 positives in one pool, 1 in other pool |
| W 12 | 1966 | 179 | | 0 | 6538 | 435 | 1+ | +positive individual could not be retrieved |
| W 13 | 18 | 2 | | 0 | 503 | 36 | 0 |  |
| W 14 | 18 | 3 | | 0 | 858 | 55 | 0 |  |
| W 15 | 5260 | 506 | | 3 | 2558 | 181 | 0 |  |
| W16 | 6338 | 571 | | 3 | 2829 | 186 | 0 |  |
| W17 | 6075 | 583 | | 3 | 3376 | 221 | 1# | # 2 positives in one pool (relatives) |
| W18 | 6822 | 613 | | 0 | 5063 | 335 | 0 |  |
| W19 | 11959 | 962 | | 1 | 5345 | 332 | 0 |  |
| W20 | 15981 | 1397 | | 4 | 6157 | 387 | 1 |  |
| **total** | **56846** | **4997** | | **15** | **44499** | **2888** | **5** |  |
